# Robust Surface Reconstruction of Plant Leaves from 3D Point Clouds

**DOI:** 10.34133/2021/3184185

**Published:** 2021-04-02

**Authors:** Ryuhei Ando, Yuko Ozasa, Wei Guo

**Affiliations:** ^1^Graduate School of Science and Technology, Keio University, Japan; ^2^School of System Design and Technology, Tokyo Denki University, Japan; ^3^International Field Phenomics Research Laboratory, Institute for Sustainable Agro-ecosystem Services, Graduate School of Agricultural and Life Sciences, The University of Tokyo, Tokyo, Japan

## Abstract

The automation of plant phenotyping using 3D imaging techniques is indispensable. However, conventional methods for reconstructing the leaf surface from 3D point clouds have a trade-off between the accuracy of leaf surface reconstruction and the method's robustness against noise and missing points. To mitigate this trade-off, we developed a leaf surface reconstruction method that reduces the effects of noise and missing points while maintaining surface reconstruction accuracy by capturing two components of the leaf (the shape and distortion of that shape) separately using leaf-specific properties. This separation simplifies leaf surface reconstruction compared with conventional methods while increasing the robustness against noise and missing points. To evaluate the proposed method, we reconstructed the leaf surfaces from 3D point clouds of leaves acquired from two crop species (soybean and sugar beet) and compared the results with those of conventional methods. The result showed that the proposed method robustly reconstructed the leaf surfaces, despite the noise and missing points for two different leaf shapes. To evaluate the stability of the leaf surface reconstructions, we also calculated the leaf surface areas for 14 consecutive days of the target leaves. The result derived from the proposed method showed less variation of values and fewer outliers compared with the conventional methods.

## 1. Introduction

Plant phenotyping is intended to provide new insights into the complex relationships between plant genotypes and phenotypes under different environmental conditions. The phenotyping is usually accomplished by determining the quantitative or qualitative values of various characteristics of the plant phenotype [1–3]. However, traditional processes for plant phenotyping are highly labor-intensive and costly [4]. Therefore, technologies for the automatic derivation of plant phenotypic traits are indispensable [5].

To automate plant phenotyping, researchers have rapidly advanced plant phenotyping techniques using imaging techniques. Among them, 3D (three-dimensional) imaging technologies have been widely applied because they can measure plant physical traits more directly than 2D (two-dimensional) imaging technologies [6]. There are two main groups of approaches to representing the 3D structure of objects [7]. First, active approaches use active sensors such as LiDAR (light detection and ranging) to directly capture a 3D point cloud that represents the coordinates of each part of the plant in 3D space [8, 9]. Second, passive approaches use passive sensors such as cameras to generate a 3D point cloud that is inferred from a set of 2D images captured from multiple perspectives [10–12].

It is necessary to convert the point cloud into a geometric representation to extract geometric characteristics of plant organs, such as the leaf size and shape. The geometric characteristics can be extracted by inferring the full surface of a 3D object from the point cloud. Surface reconstruction [13] is a typical example of this conversion process, which is frequently done when geometrical characteristics are required. (Although the approaches described in this section apply to all plant organs, we will only focus on leaves in this study.) We also describe the detailed ordinary point cloud analysis pipeline for plant phenotyping in Figure S1. This paper only focuses on the step of leaf surface reconstruction described in Figure S1-(d). The step refers to a process of generating a 3D representation of leaves, which would benefit further phenotyping tasks.

Leaf surface reconstruction is an essential 3D plant phenotyping application because of the importance of leaves in exchanges of materials and energy with the plant environment. The accuracy of geometric characteristic extraction depends on the accuracy of leaf surface reconstruction. However, the 3D point cloud generated under the growth environment is affected by sensor noise and missing points, particularly when the data is acquired under nonideal sensing conditions such as in field studies. Moreover, because plants have a complex 3D structure, it is difficult for standard scanning techniques to acquire a nonoccluded 3D point cloud for structures such as leaves that overlap with each other [14]. Such nonideal sensing conditions, exacerbated by plant properties (such as light reflection and absorption), will create sensor noise and missing points in the point cloud, leading to imperfect descriptions of the plant structure. If the leaf surface reconstruction is inaccurate, the geometric characteristic will be described inaccurately, and calculations or decisions based on that characteristic will also be inaccurate. Therefore, in this study, we aimed to develop a new leaf surface reconstruction method that is robust to noise and missing points.

The existing leaf surface reconstruction methods can be grouped into two broad categories. First, the model-free methods attempt to reconstruct a leaf surface from the 3D point cloud for a leaf in a bottom-up manner by relying on a leaf surface mesh representation, without requiring a prior mathematical model that describes the leaf. This category includes several methods, including the direct triangulation method that directly connects the points in the data to calculate the triangular mesh [15–17], Poisson surface reconstruction [18–21], nonuniform rational B-spline (NURBS) surface fitting [22–25], and locally weighted scatterplot smoothing (LOESS) [26]. These methods use local information from the point cloud to reconstruct the surface with high accuracy. However, these approaches have difficulty dealing with severe noise and missing points, since these conditions violate assumptions such as connectivity and nondegeneracy of the reconstructed 3D surface mesh [27].

To mitigate these problems with the model-free method, the second category of approaches relies on model-based methods to reconstruct the leaf surface in a top-down manner, which takes advantage of prior knowledge about the leaf that is embodied in a mathematical model [27, 28]. These methods then fit the generic model to the input data (here, the 3D point cloud) by determining the optimal values of a few parameters. Because the model can identify and decrease noise and can infer the values of missing points, it is more robust under nonoptimal measurement conditions [27]. Model-based methods can capture the morphological differences within a plant species. However, they have difficulty capturing the subtle differences within each individual leaf because of the model's generality. Also, a model that works well for one species may be unsuitable for other species, and this potentially requires the development and calibration of a new model for each species.

The model-free methods are generally more suitable for extracting detailed leaf shapes from a 3D point cloud because they detect specific features within the point cloud. However, it is difficult to apply a model-free method to 3D plant phenotyping directly. The sensitivity to noise and missing points leads to inconsistent quantification of phenotypic characteristics and high variation between measurements. For this approach to be useful in 3D plant phenotyping applications, the leaf surface reconstruction algorithm needs to output a consistent, stable, and highly accurate result from the leaf point cloud even under nonideal sensing conditions. This difficulty leads to a trade-off between the accuracy of the leaf surface reconstruction and its robustness against noise and missing points.

**Figure 1:**
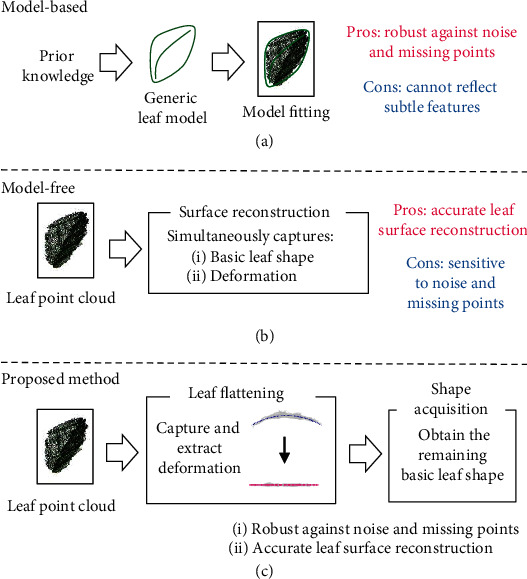
Comparison of the two traditional approaches to leaf surface reconstruction and the proposed new method: (a) the model-based method, (b) the model-free method, and (c) the proposed new method.

In this paper, we propose a new method to robustly and accurately reconstruct the leaf surface despite the noise and missing points. The process splits the leaf shape into two components: the flattened leaf shape and distortions in this shape. The flattened leaf shape refers to a shape acquired by placing the 3D leaf flatly onto a 2D plane. The distortion of the leaf shape refers to leaf bending and rolling, particularly in this paper. In the traditional model-free methods, the flattened leaf shape and its distortion are captured simultaneously by the surface reconstruction algorithm, which complicates the surface reconstruction and makes it more sensitive to noise and missing points. Instead, we assumed that its veins constrain the leaf shape distortion. Therefore, our method captures the two components of the leaf shape separately based on this assumption. This separation simplifies the surface reconstruction, thus increasing its robustness to noise and missing points.  Figure 1 shows the relationship between the methods. Our proposed method represents an approach that is intermediate between the model-free and model-based methods and takes advantage of each method's strengths. By doing so, we achieve a better trade-off between the accuracy of leaf surface reconstruction and the robustness against noise and missing points.

To evaluate the effectiveness of the proposed method, we reconstructed the leaf surface from two species of the crop: soybean (*Glycine max*) and sugar beet (*Beta vulgaris*). We split the leaf point cloud data into four datasets: two levels of missing points in the point cloud × two species. We compared the accuracy and robustness of the proposed method with those of the model-free methods. We excluded model-based methods from the evaluation because they cannot capture the leaf surface in detail due to the generality of the mathematical model. We confirmed that the proposed method stably and robustly reconstructs the leaf surface for a range of noise levels and numbers of missing points for two species with different leaf shapes. To evaluate the stability of the leaf surface reconstructions, we also grew sugar beet leaves for 14 days and calculated the reconstructed leaf surface area. We confirmed that the leaf surface area derived using the proposed method shows less variation and fewer outliers than the model-free method and is thus more stable.

## 2. Materials and Methods

### 2.1. Experimental Design and 3D Point Cloud Acquisition

We conducted our greenhouse experiment with two species (sugar beet and soybean) at the Institute for Sustainable Agro-ecosystem Services, The University of Tokyo (35°44′22^″^N, 139°32′34^″^E and 60 m above sea level), under natural light conditions. To obtain a diversity of leaf shapes, we chose three genotypes of sugar beet (NK195BR, NK388, and NK195BR × NK388) and three soybean genotypes (Peking, Enrei, and Williams 82) and planted them in separate containers (at 1 container per genotype). The containers were automatically irrigated to field capacity at intervals of 1 day.

We obtained the point clouds using a 3D imaging system developed by Phenospex (Heerlen, the Netherlands), in which the system moves two PlantEye F500 laser light section scanners over the plant to record multispectral data (red, green, blue, and near-infrared light). The sugar beet plants were transplanted into containers on 21 August 2017 and were continuously scanned from 24 August to 7 September. The average photoperiod is 12 h, photosynthetic photon flux density (PPFD) is 837~1023 *μ*mol m^−2^ s^−1^ with an average of 969 *μ*mol m^−2^ s^−1^, relative humidity ranged from 52% to 93% with an average of 77%, and temperatures ranged from 21°C (night) to 33°C (day) with an average of 26°C on 1 September 2017. The scan generated a total of 1102 files in the polygon (∗.ply) format. The soybean plants were sowed directly on 17 April 2019 and continuously scanned from 23 April to 27 May 2019. The average photoperiod is 13 h, photosynthetic photon flux density (PPFD) is 780~1023 *μ*mol m^−2^ s^−1^ with an average of 991 *μ*mol m^−2^ s^−1^, relative humidity ranged from 24% to 96% with an average of 58%, and temperatures ranged from 10°C (night) to 37°C (day) with an average of 22°C on 10 May 2019. The scan generated a total of 17 files in the ∗.ply format.

### 2.2. Point Cloud Preprocessing

During preprocessing, we statistically removed noise from the raw point cloud. We performed the noise removal using the statistical outlier removal filter implemented in the Point Cloud Library (PCL) software ([29]). The raw point cloud consists of sampled points of leaves with 3 dimensions, *x*-, *y*-, and *z*-axis coordinates. The *z*-axis is aligned along the direction vertically upward with respect to the ground.

We manually performed leaf segmentation. To do so, we defined a threshold for the points along the *z*-axis to exclude points that did not belong to the leaf. We defined the threshold value at the soil surface. We then selected a leaf to segment and manually searched for a 2D image plane that completely separated the leaf from the other leaves. Next, we projected the leaf point cloud onto the 2D image plane to create a polygon that fully included the point cloud for the selected leaf [30]. The polygon was created by manually generating vertex points within the 2D image plane. Vertices were chosen as close as possible to the outermost points in the 2D plane. Finally, we reprojected the point cloud inside the polygon back into the 3D space to identify each 2D point's original point in the cloud. To segment the leaf in time sequence data of sugar beet, we conducted the segmentation process frame by frame throughout the data sequence from frame-0 to frame-1102 to obtain the leaf point cloud in time series.

To provide a consistent reference system, we transformed the origin of each leaf with respect to the position where the plant emerged from the soil, which we determined manually. The leaves therefore had the same origin if they belonged to the same plant.

The whole process of noise removal and manual leaf segmentation was implemented in C++ and PCL.

### 2.3. Proposed Method

We split the shape of the leaf into two components: the flattened leaf shape and its distortion. The distortion of the leaf shape will be leaf bending and rolling, particularly in our paper. The flattened leaf shape is the shape we obtain when we flatten the leaf on a flat table. We assumed that the distortion of the flattened leaf shape was constrained by the midrib/primary vein and secondary veins. We captured the two components of the leaf shape separately using this constraint.

**Figure 2:**
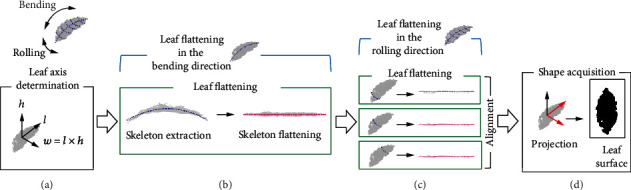
Overview of the method used to obtain the flattened leaf shape: (a) leaf axis determination (note that the equation is for a vector cross product, not simple multiplication), (b) leaf flattening in the bending direction, (c) leaf flattening in the rolling direction, and (d) shape acquisition.

Our method extracts the distortion along two axes: with respect to the direction along the leaf's primary vein (the bending direction) and with respect to the direction along the leaf's secondary veins (the rolling direction). As a result, we obtain a leaf shape with the distortion removed, which we used as the flattened leaf shape. The input for this analysis is the 3D point cloud, and the output is a 2D point cloud.  Figure 2 summarizes the workflow. Details of these steps are presented in the rest of Section 2.3.

The workflow comprises four steps: determination of the two leaf axes (bending with respect to the primary vein and rolling with respect to the secondary veins; Figure 2(a)), flattening in the bending direction (Figure 2(b)), flattening in the rolling direction (Figure 2(c)), and shape acquisition (Figure 2(d)). The flattening accounts for 3D distortion of the leaf surface and is performed by extraction of the skeleton (the primary and secondary veins), followed by flattening of the skeleton. We implemented the algorithm for the proposed method in Python (http://www.python.org/). The source code is at https://github.com/oceam/LeafSurfaceReconstruction.

#### 2.3.1. Leaf Axis Determination

To consistently model the leaf point cloud, we first defined three axes that together represent the leaf: the length axis defined by the direction of the primary leaf vein (the *l*-axis), the axis that is perpendicular to the leaf surface (the *h*-axis), and the axis that is perpendicular to both these axes (the *w*-axis). We defined each leaf coordinate system based on these axes. This lets us determine the bending and rolling directions of the leaf, regardless of its orientation in 3D space. Algorithm S1 describes the process.

To obtain the 3D unit direction vector **l** ∈ ℝ^3^ for the *l*-axis that consistently points from the leaf's base to the leaf's apex, we first applied classical principal component analysis to the input 3D leaf point cloud *P*_init_ = {**p**_init,*i*_ ∈ ℝ^3^ | 0 ≤ *i* ≤ *M* − 1}. The 3D point cloud is a set of points with its coordinates in 3D space. We denote **pc**_1_ as the eigenvector corresponding to the largest eigenvalue of the covariance matrix of *P*_init_ in 3D space. Here, we ensure that the vector toward the centroid of *P*_init_ from the origin and the direction vector **l** point in the same direction.

(1) $$\begin{array}{c} \phi =\arccos\left(\frac{c∙pc_{1}}{\left|c\right|\left|pc_{1}\right|}\right),\\ l=\left\{\begin{array}{c} \;pc_{1},\;\;\phi \leq \frac{\pi }{2},\\ -pc_{1},\;\text{ otherwise}, \end{array}\right. \end{array}$$

where **c** is the centroid of *P*_init_. In this paper, we assume that the leaf length along the leaf vein direction is longer than the leaf width. Once the unit direction vector **l** is determined, we shift the origin of *P*_init_ to its centroid for further processing.

For the *h*-axis, we want it to represent the top view of the leaf. **pc**_3_, which is the eigenvector corresponding to the smallest eigenvalue of the covariance matrix of *P*_init_, cannot be used directly for the *h*-axis when the distortion of the leaf is large toward the bending direction along the leaf vein. This is because, as the distortion gets larger, the variance in the data gets larger toward the direction the leaf is bending. This occasionally leads **pc**_3_ to point toward the side of the leaf, which we cannot use directly for the *h*-axis. Instead, we searched for the plane that represents the view from the side of the leaf and used the direction vector perpendicular to this plane. In this way, we can consistently determine the *h*-axis of the leaf point cloud even when the leaf contains substantial distortion.

**Figure 3:**
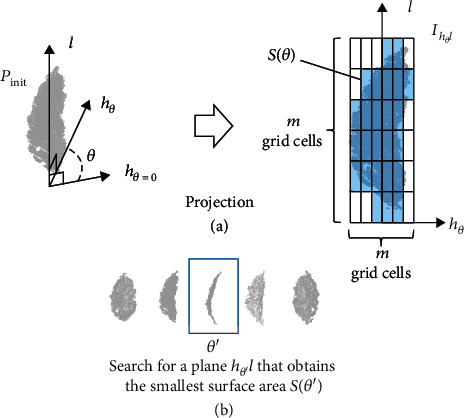
Illustration of the process for leaf axis determination. (a) Calculation of the projected area. The grid cells in which points exist are shaded blue, whereas the actual leaf outline is shown in dark blue. (b) Search for the minimum projected area.

Figure 3 illustrates the search process for the plane representing the side view of the leaf. First, we randomly generated an initial axis *d* that was perpendicular to the *l*-axis. We rotated this *d*-axis around the *l*-axis for *θ* (*θ* = 0°, 1°, ⋯, 180°) and created the *h*_*θ*_-axis. Note that *h*_*θ*=0_ = *d*. We then projected the point cloud on the *h*_*θ*_*l*-plane to generate a 2D binary image *I*_*h*_*θ*_*l*_ by creating a rectangular grid over the *h*_*θ*_*l*-plane. To determine the grid, we selected two points in *P*_init_ with the minimum and maximum *l*-axis element values, respectively. We then split the section between the two points to create *m* equally spaced sections. We created another *m* equally spaced sections by performing the same calculation against the *h*_*θ*=0_-axis direction and combined the sections to create a *m* × *m* rectangular grid. *m* was set to 100 in the experiment, since there was only a slight improvement by increasing *m* from 100 in our settings. Each grid cell in this rectangular grid corresponded to each pixel in *I*_*h*_*θ*_*l*_. Note that the rectangular grid was determined only once in this leaf axis determination process. The pixel value of *I*_*h*_*θ*_*l*_ will be 1 if a point exists inside the rectangular grid cell and 0 otherwise. The projected surface area *S*(*θ*) could be calculated from *I*_*h*_*θ*_*l*_ by totaling the values (0 or 1) in each pixel in *I*_*h*_*θ*_*l*_ (Figure 3(a)). This process was repeated by rotating the *h*_*θ*_-axis around the *l*-axis for every *θ*. Finally, we obtained an *h*_*θ*′_*l*-plane with the minimum surface area, which was *S*(*θ*′), and we utilized this *h*_*θ*′_-axis as the *h*-axis (Figure 3(b)).

The determined *h*-axis might point vertically downwards, depending on the view of the leaf that was projected. In that case, we reversed the *h*-axis direction vector so that every *h*-axis pointed away from the ground. Finally, we determined the *w*-axis by computing the cross product of the direction vectors for the *l*-axis and *h*-axis:

(2) $$\begin{array}{c} w=l\times h, \end{array}$$

where **w** ∈ ℝ^3^ was the 3D unit direction vector for the *w*-axis, and **h** ∈ ℝ^3^ was the 3D unit direction vector for the *h*-axis.

Once we had defined all three leaf axes, we transformed the input 3D leaf point cloud *P*_init_ from the original (*x*, *y*, *z*) coordinate system to the (*l*, *w*, *h*) leaf coordinate system to determine the transformed 3D leaf point cloud *P* = {**p**_*i*_ ∈ ℝ^3^ | 0 ≤ *i* ≤ *M* − 1}.

#### 2.3.2. Skeleton Extraction

To capture the shape distortion, we extracted a curved skeleton (the primary and secondary veins) from the leaf point cloud. This process takes the 3D point cloud *Q* = {**q**_*i*_ ∈ ℝ^3^ | 0 ≤ *i* ≤ *M* − 1} and outputs the skeleton points *S* = {**s**_*i*_ ∈ ℝ^2^ | 0 ≤ *i* ≤ *N* − 1}. Inspired by the manifold projection procedure in [31], we projected the point cloud *Q* onto the 2D plane and performed curve fitting. From the fitted curve, we sampled a 2D point that represented the distortion. We used this process to estimate the distortion of the leaf that must be removed during the skeleton flattening process described in Section 2.3.3. Algorithm S2 describes this process. We explain the detail of this process below.

First, we tested whether the 3D point cloud *Q* had enough points to extract a skeleton to ensure that the skeleton points *S* will be extracted with acceptable accuracy. We did not process the point cloud if the number of points in *Q* was less than *δ* points. We set the number of points *δ* for discarding the leaf points to 5 to obtain more reliable results than the result only using the least number of points for fitting a cubic function, which is 3.

Second, we selected two axes from the *w*-, *h*-, and *l*-axes to create a 2D plane to project the point cloud. We called this plane a skeleton plane. Here, we denoted the selected horizontal axis *a* and vertical axis *b*. For example, we choose *l* as *a* and *h* as *b* for flattening in the bending direction and *w* as *a* and *h* as *b* for flattening in the rolling direction. Then, we projected the leaf point cloud *Q* onto the *ab*-plane and fit a skeleton function *f* by means of least square linear regression.

Finally, we uniformly sampled *N* points between the minimum and maximum values of the *a*-axis element of the points and defined these points as *U* = {**u**_*i*_ ∈ ℝ^1^ | 0 ≤ *i* ≤ *N* − 1}, and we computed the skeleton points *S* by

(3) $$\begin{array}{c} S=f\left(U\right). \end{array}$$

Exceptionally, we expanded this section of sampling *U* for 5% only for the skeleton extraction in the bending direction. The reason for this expansion is that we observed some leaves (mostly sugar beet) with their tips heavily curled up, and there was a need to expand the sampling section to visualize the whole leaf. We set the number of sampled points *N* to 50 in the experiment. We set the skeleton function *f* to quadratic function for the skeleton extraction in the bending direction with reference to the manifold projection procedure [31]. We set *f* to cubic function for the skeleton extraction in the rolling direction since the leaves we used for the experiment contained more complex distortion in the rolling direction than the bending direction. Note that the points in *U* were indexed in order from the minimum to maximum values along the *a*-axis, so that the skeleton points *S* were indexed in the same order.

#### 2.3.3. Skeleton Flattening

**Figure 4:**
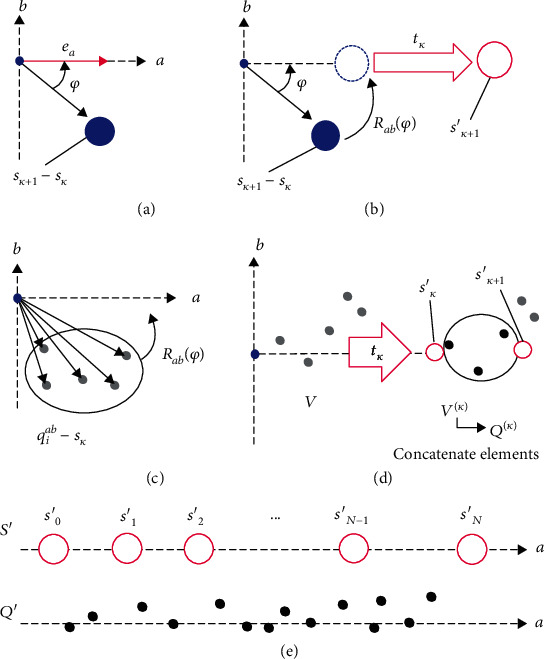
Illustration of the steps in the skeleton flattening process. (a) Calculation of the angle between the *a* point and the *a*-axis. (b) Flattening of the skeleton. (c) Rotation of the leaf point cloud. (d) Extracting strips from the leaf point cloud. (e) Final results of the flattening process.

To remove distortion from the leaf point cloud, we flattened the distortion extracted by the skeleton extraction step in Section 2.3.2 by rotating the points in the leaf point cloud that existed between two skeleton points and then moving them along the axis in the direction required to eliminate the distortion. By removing the distortion of the leaf point cloud, we could extract the flattened leaf shape from the output. This process takes the skeleton points *S* and the leaf point cloud *Q* as inputs and outputs the flattened skeleton points *S*′ = {**s**′_*i*_ ∈ ℝ^2^ | 0 ≤ *i* ≤ *N* − 1} and the flattened leaf point cloud *Q*′ = {**q**′_*i*_ ∈ ℝ^3^ | 0 ≤ *i* ≤ *M* − 1}. Flattening was done by rotating the points that existed between each skeleton point and arranging them along the straight line. The skeleton plane *ab*, which was used to determine the skeleton point *S*, was used again during this process.  Figure 4 illustrates the stages involved in flattening the leaf skeleton point cloud. Algorithm S3 describes the procedure.

First, we initialized the flattened skeleton point by setting **s**′_0_ to the origin of the coordinates, and then we calculated the angle *φ* (−*π* < *φ* < *π*) between the *k* + 1-th skeleton point **s**_*k*+1_ and the *a*-axis (Figure 4(a)). We calculate *φ* by solving

(4) $$\begin{array}{c} \varphi =\arctan\left(-\frac{\left(s_{k+1}-s_{k}\right)∙e_{b}\;}{\left(s_{k+1}-s_{k}\right)∙e_{a}}\right), \end{array}$$

where **e**_*a*_ ∈ ℝ^2^ and **e**_*b*_ ∈ ℝ^2^ are the unit basis vectors for the *a*-axis and *b*-axis, respectively.

Second, we used this value of *φ* to rotate **s**_*k*+1_ onto the *a*-axis, and we translated it along the *a*-axis by a distance equal to the scalar value *t*_*k*_, which was defined as

(5) $$\begin{array}{c} t_{k}=\left\{\begin{array}{c} 0,\;k=0,\\ \left\|s_{k}^{\prime }-s_{0}^{\prime }\right\|,\;\;1\leq k\leq N-2. \end{array}\right. \end{array}$$

The flattened skeleton point **s**′_*k*+1_ could be determined by

(6) $$\begin{array}{c} s_{k+1}^{\prime }=R_{ab}\left(\varphi \right)\left\{s_{k+1}-s_{k}\right\}+t_{k}e_{a}, \end{array}$$

where **R**_*ab*_(*φ*) is a two-dimensional rotation matrix for rotating a 2D vector around the origin of the *ab*-plane by *φ* (as shown in Figure 4(b)).

As we flattened the skeleton points, we performed the flattening of the leaf point cloud *Q*. We projected all the points in the leaf point cloud *Q* onto the *ab*-plane and generated the 2D point cloud *Q*^*ab*^ = {**q**_*i*_^*ab*^ ∈ ℝ^2^ | 0 ≤ *i* ≤ *M* − 1}. Note that the elements corresponding to the axis in *Q* that was not used for the skeleton plane were restored later (as described in Figure 4(d)). We performed a similar calculation for the 2D point cloud *Q*^*ab*^ that was done in equation (6) to rotate the points around the origin of the *ab*-plane by *φ* (Figure 4(c)) and translated it along the *a*-axis by a distance equal to the scalar value *t*_*k*_ (Figure 4(d)) to determine the translated 2D point cloud *V* = {**v**_*i*_ ∈ ℝ^2^ | 0 ≤ *i* ≤ *M* − 1} by

(7) $$\begin{array}{c} v_{i}=R_{ab}\left(\varphi \right)\left\{q_{i}^{ab}-s_{k}\right\}+t_{k}e_{a}. \end{array}$$

Here, we sampled only the points from *V* that existed between the flattened skeleton points **s**′_*k*_ and **s**′_*k*+1_ on the *a*-axis (Figure 4(d)). By this process, we could arrange the point cloud little by little, finally resulting in a whole leaf point cloud that was aligned straight along the *a*-axis. We denoted the point cloud of *M*_*k*_ sampled points between **s**′_*k*_ and **s**′_*k*+1_ as *V*^(*k*)^ = {**v**_*i*_^(*k*)^ ∈ ℝ^2^ | 0 ≤ *i* ≤ *M*_*k*_ − 1}. Finally, we removed and concatenated the element of the point in the leaf point cloud *Q*, the element corresponding to the axis that was not used for the skeleton plane, to each corresponding point in *V*^(*k*)^ and generated the strip leaf point cloud *Q*^(*k*)^ = {**q**_*i*_^(*k*)^ ∈ ℝ^3^ | 0 ≤ *i* ≤ *M*_*k*_ − 1}. For example, if the *lh*-plane was the skeleton plane, we removed the *w*-axis elements from the points in the leaf point cloud *Q* to generate *Q*^(*k*)^ from *V*^(*k*)^. We repeated the procedure of generating the *P*^(*k*)^ for every *k* = 0, ⋯, *N* − 2. We gathered all the points in *Q*^(*k*)^ for every *k* and defined the flattened leaf point cloud as *P*′. The final outputs of the flattened skeleton point cloud *S*′ and the flattened point cloud in *P*′ were arranged directly along the *a*-axis, as shown in Figure 4(e).

#### 2.3.4. Leaf Flattening in the Bending Direction

To capture and remove distortion in the bending direction along the leaf veins, we performed the leaf flattening process along this axis. We set the *lh*-plane to be the skeleton plane and performed the leaf flattening process using the transformed 3D leaf point cloud *P*. We obtained the 3D leaf point cloud *P*′ that had been flattened in the bending direction in which the points were arranged along the *l*-axis. Here, we save the strip leaf point clouds {*P*^(0)^, ⋯, *P*^(*N* − 1)^} that would be calculated during the skeleton flattening process and used them as the input for leaf flattening in the rolling direction, which is described in the next section.

#### 2.3.5. Leaf Flattening in the Rolling Direction

**Figure 5:**
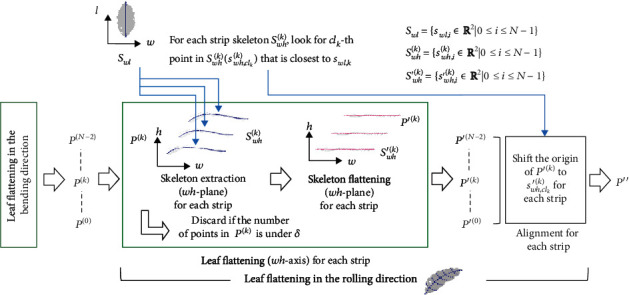
Illustration of the workflow for flattening in the rolling direction.

We also extracted and flattened the distortion in the rolling direction along the leaf veins. We used the strip leaf point clouds {*P*^(0)^, ⋯, *P*^(*N* − 1)^} from leaf flattening in the bending direction as inputs. We set the *hw*-plane as the skeleton plane and performed the leaf flattening process for every strip in the leaf point cloud.  Figure 5 illustrates the workflow. By applying the skeleton extraction to each strip in the leaf point cloud *P*^(*k*)^, we obtained the strip skeleton points *S*_*wh*_^(*k*)^ = {**s**_*wh*,*i*_^(*k*)^ ∈ ℝ^2^ | 0 ≤ *i* ≤ *N* − 1}. Taking the strip leaf point cloud *P*^(*k*)^ and the strip skeleton point cloud *S*_*wh*_^(*k*)^ as inputs, we performed the skeleton flattening process in the rolling direction and obtained the flattened strip leaf point cloud *P*^′(*k*)^ and the flattened strip skeleton point cloud *S*_*wh*_^′(*k*)^ = {**s**_*wh*,*i*_^′(*k*)^ ∈ ℝ^2^ | 0 ≤ *i* ≤ *N* − 1}.

We gathered the points of every flattened strip in the leaf point cloud *P*^′(*k*)^ to create a 3D point cloud *P*′′ that had been flattened in both the bending direction and the rolling direction. However, simply gathering the points would have resulted in a collapsed leaf surface because every *P*^′(*k*)^ was aligned with respect to the origin of the *w*-axis. This resulted from the initialization step during the skeleton flattening procedure (from equation (5), *k* = 0). To overcome this problem, we calculated alignment skeleton points *S*_*wl*_ = {**s**_*wl*,*i*_ ∈ ℝ^2^ | 0 ≤ *i* ≤ *N* − 1} and aligned them to *P*^′(*k*)^ with this skeleton. To compute *S*_*wl*_, we set the skeleton plane to the *wl*-plane and performed the skeleton extraction procedure for the 3D leaf point cloud *P*.

To align with *S*_*wl*_, we used the strip skeleton points *S*_*wh*_^(*k*)^. For each strip skeleton *S*_*wh*_^(*k*)^, we looked for a *cl*_*k*_-th point in *S*_*wh*_^(*k*)^, which is **s**_*wh*,*cl*_*k*__^(*k*)^, for which the distance in the 3D space was closest to the *k*-th point in the alignment skeleton points *S*_*wl*_, which is **s**_*wl*,*k*_. By this process, we found the position of a landmark point that could be used to align the points in *P*^′(*k*)^. We then extracted the landmark point by removing the *cl*_*k*_-th point from the flattened strip skeleton points *S*_*wh*_^′(*k*)^, which is **s**_*wh*,*cl*_*k*__^′(*k*)^. We concluded this step by shifting the origin of *P*^′(*k*)^ to the landmark point **s**_*wh*,*cl*_*k*__^′(*k*)^. Note that the landmark point **s**_*wh*,*cl*_*k*__^′(*k*)^ was a 2D point on the *wh*-plane, so we added a dimension corresponding to the *l*-axis and set its value to 0 before shifting the origin. We repeated the alignment procedure for every *k*. After the alignment, we gathered all the points in the strip into the leaf point cloud *P*′′.

#### 2.3.6. Shape Acquisition

At this point in the analysis, we had removed distortion in the bending and rolling directions along the leaf veins. As a result, the leaf point cloud *P*′′ contained only the flattened leaf shape. The leaf point cloud was flattened in the *wl*-plane, so we simply projected *P*′′ onto the *wl*-plane to obtain the leaf surface. The final output is the 2D point cloud after the projection. Note that we have computed skeleton points for this point cloud; thus, we obtain the shape by *P*′′ and skeleton point clouds computed from the two leaf flattening steps.

### 2.4. Evaluation of the Robustness of the Leaf Surface Reconstruction

We evaluated our method in terms of the robustness of the leaf surface reconstruction against noise and missing points. We compared the leaf surface reconstruction results of the proposed method with Poisson surface reconstruction [32] and NURBS surface fitting [33]. Also, we added the comparison with the “Surface Reconstruction” step from their full process pipeline in Zhu et al. [26] as the reference. We excluded the triangulation method that directly computes the triangular mesh from the data from the comparison because it utilized the noisy leaf point cloud directly to define the vertices of triangles and create the mesh surface. We assumed that data gathered during measurement of the plant included sensor noise and missing points, and the direct triangulation method is known [32, 34] not to be robust against noise and missing points. We also excluded model-based methods from the comparison because they assumed a preexisting model rather than generating the plant leaf surface solely from a real plant. The model-based methods should not be considered for us because they can only build the leaf shape with predefined knowledge.

Although model-free methods have high sensitivity to the quality of the point cloud, they can tune the hyperparameters of the surface reconstruction to lower its sensitivity to noise and missing points. Therefore, we compared the results produced by our new method using high and low sensitivities for its hyperparameter settings. For the screened Poisson surface reconstruction, we used the implementation from Kazhdan and Hoppe [32]. We used the default parameter value from their implementation as the high sensitivity setting. For the low sensitivity setting, we changed the parameter that determines the maximum depth of the tree that will be used in the algorithm (the reconstruction depth) from 8 to 6. In the reconstruction, it was necessary to exclude surface regions that lay outside the shape defined by the majority of the points. We set the threshold value for mesh trimming (the trimming value) to 6 for sugar beet leaves and to 5 for soybean leaves empirically. For the NURBS surface fitting, we used the C++ implementation in PCL, as described in Santos et al. [22]. We changed the number of refinement iterations from 5 to 2 and the number of iterations that are performed after the refinement from 10 to 2 to reduce the computational cost. We used this setting as the high sensitivity setting. For the low sensitivity setting, we changed the refinement iterations to 1. For Zhu et al.'s method, we used the same configuration as in the paper [26].

The reconstruction results from our model differed from the results of a model-free method that contained both elements of the leaf shape, because our method contained only the flattened leaf shape and extracted the distortion separately. We could flatten the results of the other model-free methods by applying the UV flattening algorithm [35] to convert the 3D surface to a 2D surface and allow a comparison on the 2D plane, but this would have deformed the shape of the leaf surface. Thus, it was difficult to directly compare the methods. For this reason, we instead projected the results of all model-free methods on the plane that we used for flattening the leaf (the *wl*-plane) before evaluating the robustness of the methods.

When used in plant phenotyping applications, leaf surface reconstruction is sensitive to differences in the leaf shape both within and between species. The amount of noise and number of missing points that result from the nonideal sensing conditions and the complex structure of plants may also differ. To evaluate the effectiveness of our method under a range of conditions, we analyzed the point cloud data separately for the two species (soybean and sugar beet) and for the different numbers of missing points in the point cloud (which we determined visually). We did not specifically account for noise because all of the data contained sensor noise to some extent. The missing points will be caused by sensing conditions or occlusions. Occlusion includes the self-occlusion which refers to the occlusion caused by the leaf itself or overlapping (Figure S2). For each analysis condition, we manually selected 2 leaves from the data. The resulting division of the data produced a total of four datasets.

The experiment done in this paper (Sections 2.4–2.6) focuses only on the leaf surface reconstruction part and not the complete pipeline for 3D plant phenotyping, since we assume that the preprocesses are done before applying the proposed method.

### 2.5. Evaluation of the Leaf Surface Reconstruction Stability

We evaluated our method in terms of the stability of the leaf surface reconstruction to support plant phenotyping. We extracted and plotted the dynamics of the leaf surface area from the 3D point cloud acquired during plant development. For the model-free methods, we used the low sensitivity setting to minimize the effect of noise and missing points in the point cloud data.

We calculated the leaf area from the leaf surfaces reconstructed from the 1102 frames of leaf point cloud data for sugar beet, with point data obtained at 20-minute intervals for 14 days. We set the sensitivity setting to low. In our method, we calculated the rectangular area for each flattened strip leaf point cloud *P*′^(*k*)^ that was determined during the leaf flattening step in the rolling direction and summed the values for all strips. To calculate the rectangular area, we utilized the flattened skeleton points *S*_*lh*_′ = {**s**′_*lh*,*i*_ ∈ ℝ^2^ | 0 ≤ *i* ≤ *N* − 1} calculated during the leaf flattening step for the bending direction. We used the distance between adjacent points in *S*_*lh*_′ as the sides of the rectangles. The reconstructed leaf area *S*_rec_ was calculated as follows:

(8) $$\begin{array}{c} S_{\text{rec}}=\overset{N-2}{\underset{k=0}{\sum }}\left|w_{\max}^{k}-w_{\min}^{k}\right|\left|s_{lh,k+1}^{\prime }-s_{lh,k}^{\prime }\right|, \end{array}$$

where *w*_max_^*k*^ and *w*_min_^*k*^ represent the maximum and minimum values of the *w*-axis elements of *P*′^(*k*)^.

We plotted the leaf area value from each frame and used the sequence of areas to quantify the stability of the leaf surface reconstruction. The surface area of plant leaves should change continuously within each phenological stage, so values that differed dramatically between adjacent frames indicate an undesirable result. We also conducted a regression analysis to determine whether the leaf areas follow a statistically linear equation. We compute the *R*^2^ score from this linear function to evaluate the stableness of the method. By comparing the *R*^2^ score, we evaluate how far the values are away from the fitted linear function, thus evaluating the stability of the method in time series.

### 2.6. Quantification of the Degree of Distortion

We quantify the degree of distortion from the results of the leaf surface calculated by the proposed method to verify the effectiveness of leaf flattening. To quantify the degree of distortion, we calculated the mean and standard deviation of the distance from the 2D projected surface (*wl*-plane). To calculate the distance from the *wl*-plane, we simply used the *h*-axis element values. We calculated the degree of distortion from the leaf surfaces reconstructed by our method from the dataset we used to evaluate the robustness and stability of leaf surface reconstruction in Sections 2.4 and 2.5.

## 3. Results

### 3.1. Evaluation of the Robustness of the Leaf Surface Reconstruction

**Figure 6:**
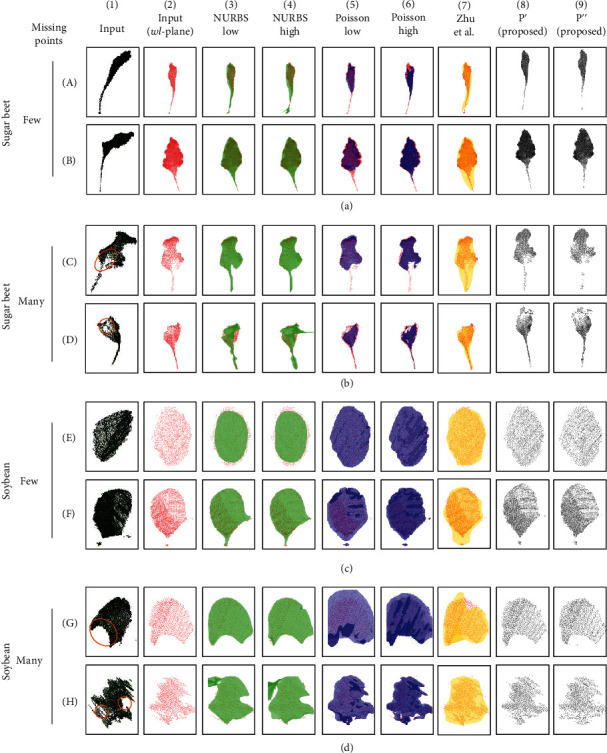
Results of the evaluation of the leaf surface reconstruction in terms of robustness against noise and missing points. Each row (A–H) corresponds to the input point cloud for one leaf. Each pair of rows ((a) plus (b), (c) plus (d)) for a species corresponds to a split dataset with few and many missing points. Column 1 corresponds to the raw data for the input leaf point cloud, column 2 corresponds to the leaf point cloud transformed to the leaf coordinate system, columns 3–7 correspond to the results from two previous model-free methods (nonuniform rational B-spline (NURBS), Poisson, and methods from Zhu et al. [26]), column 8 corresponds to the results from the flattening in the bending direction developed in the present study, and column 9 corresponds to results from the flattening in both the bending and rolling directions developed in the present study. Orange circles indicate where the missing points mostly exist in the leaf point cloud.

Figure 6 illustrates the input data and the leaf surfaces produced by the three models, with few and many missing points. Each result was projected onto the *wl*-plane that we determined during the leaf axis determination process. For each leaf surface, we have provided an indication of the positions of the corresponding input leaf cloud in red.

The reconstruction results from the NURBS surface fitting (Figure 6, columns 3 and 4) tended to contain overestimates, which represented where the produced surface overfits the point cloud data for a leaf surface (for example, the result located at row A, column 4). Some of the results produced surfaces with twists (the result located at row D, columns 3 and 4) and jagged outlines (the result located at row H, columns 3 and 4) by severely overestimating the input data. There were also results that contain underestimates, which represented where the produced surface underfits the point cloud data (for example, the result located at row E, column 3). From both datasets with few and many missing points, the reconstruction lost shape information. However, some of the images provided a good match to the input point cloud data (for example, the result located at row C, column 4).

The results from the Poisson surface reconstruction (Figure 6, columns 5 and 6) tended to contain bumpy surfaces that overestimated the point cloud data (for example, the result located at row F, columns 5 and 6). This trend was mostly seen in the soybean. Instead of the sugar beet, it tended to contain missing surfaces, which represented where the surface was not reconstructed with the existing leaf point cloud (for example, the result located at rows C and D, columns 5 and 6). From both datasets with few and many missing points, the reconstruction lost shape information, but some of the results provided a good match to the input point cloud data (for example, the result located at row E, column 6).

In contrast, our new method outputs a leaf surface (Figure 6, columns 8 and 9) without losing too much of the shape information and without adding artifacts such as twists (for example, the result located at row D, column 4), jagged outlines (for example, the result located at row H, columns 3 and 4), bumps (for example, the result located at row F, columns 5 and 6), or missing parts of the surface (for example, the result located at rows C and D, columns 5 and 6). The particular example of these artifacts generated by the model-free methods is extracted and shown in Figure S3.

In addition, we have added the result of the leaf surface reconstruction step of Zhu et al. [26] from their full process pipeline as the reference. The results from Zhu et al.'s method (Figure 6, column 7) showed the best fit in the model-free method throughout the data. The bumpy surfaces or jagged outlines are not observed, which can be found from the NURBS and Poisson methods. However, it tended to overestimate the point cloud data (for example, the result located at rows B, C, and F, column 7) for both species. It showed some robustness against the leaf with severe missing points and noise (for example, the result located at row H, column 7). Overall, from both datasets with few and many missing points, the reconstruction lost shape information, but some of the results provided a good match to the input point cloud data (for example, the result located at row E, column 7).

### 3.2. Evaluation of the Leaf Surface Reconstruction Stability

**Figure 7:**
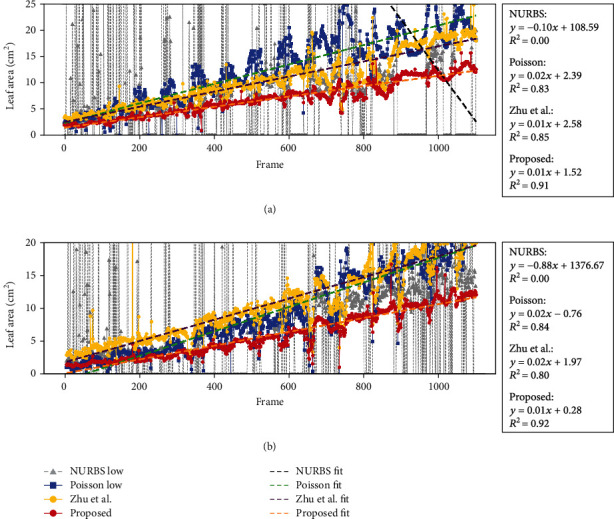
Results of the evaluation of stability for leaf surface reconstruction. The plot of the leaf area for each frame is shown on the left, and its regression analysis is shown on the right. (a, b) Represent two different sugar beet leaves. NURBS represents the nonuniform rational B-spline surface fitting method, Poisson represents the screened Poisson surface reconstruction method, and the sensitivity was set to low for both methods. Zhu et al. represent the method from [26], and “Proposed” represents the method developed in the present study.

Figure 7 shows the changes in leaf areas for two sugar beet leaves.  Table 1 shows the calculated mean and standard deviation for the difference in leaf areas between consecutive frames. The leaf area values for the NURBS surface fitting exhibited high variation, including 0 values, in both leaves. The leaf area values in the Poisson surface reconstruction showed lower but still relatively high variation common from frame-200 to frame-1100 for the first leaf (Figure 7(a)) and from frame-300 to frame-1100 for the second leaf (Figure 7(b)). The leaf area values in Zhu et al.'s method showed a similar trend to the Poisson method, with high variation common from frame-700 to frame-1100 for the first leaf (Figure 7(a)) and from frame-650 to frame-1100 for the second leaf (Figure 7(b)). In contrast, our new method showed smoother, nearly continuous changes in leaf areas between adjacent frames, with smaller variation than in the other model-free methods. The regression analysis showed that leaf area data calculated from the leaf surface produced by our method follows a statistically linear equation with a higher *R*^2^ score than the model-free methods (Figure 7, right, 0.91 for the first leaf and 0.92 for the second leaf). The comparison of the *R*^2^ value between the methods demonstrates the stability for calculating the leaf area values of the same leaf in the time series order, thus showing the stability of our leaf surface reconstruction method.

### 3.3. Quantification of the Degree of Distortion

Table 2 shows the degree of distortion calculated from the data of 8 leaves which was used to evaluate the robustness of surface reconstruction in Section 3.1 Overall, the sugar beet leaves contained more distortions compared to soybean leaves since the mean and standard deviation of the distance from the *wl*-plane were relatively higher. The values successfully decrease as the flattening procedure processes from the input 3D leaf point cloud *P* to the 3D point cloud *P*′′ flattened in both the bending direction and the rolling direction in both species. The values of *P*′′ for leaves with few missing points were relatively lower than those for leaves with many missing points in most of the cases.

Table 3 shows the degree of distortion calculated from the data of 2 sugar beet leaves in series which was used to evaluate the stability of leaf surface reconstruction in Section 3.2. The values decrease as the flattening procedure processes, indicating that our method is effectively extracting the distortion and flattening the leaf point cloud.

## 4. Discussion

### 4.1. Leaf Surface Reconstruction

The trade-off between the accuracy of leaf surface reconstruction and the robustness against noise and missing points is the main difficulty when reconstructing a leaf surface from a 3D point cloud to support plant phenotyping. The new leaf surface reconstruction method developed in the present study separately captured the flattened leaf shape and its distortions by using leaf-specific properties. The new method reconstructs a leaf surface that is more robust against noise and missing points than the two previous model-free methods. This improvement appears to have resulted from our simplification of the leaf surface reconstruction process and the greater robustness of our method compared to the previous model-free methods.

The NURBS surface reconstruction [33] (implementation in [29]) results contained many overestimates. (Figure 6, columns 2 and 3). This was likely due to the complexity of the objective function used in the NURBS surface fitting algorithm. The NURBS surface was determined by its control points and weighting factors, and the method required suitable parameterization of weights and optimal numbers and positions of control points to define the surface topology. This increased the method's complexity and the difficulty of fitting the surface. Thus, it requires clean data with little noise and few missing points. When NURBS fitting was applied to real data with high noise and many missing points, the objective function reacted sensitively to these problems.

The Poisson surface reconstruction [32] tended to have bumpy surfaces for soybean and missing parts for sugar beet (Figure 6, columns 4 and 5). The Poisson objective function was also complex, and this made it react sensitively when applied to real data. It approximated an indicator function that separates points that lie inside and outside the 3D model of the leaf shape, which made it more sensitive to sensor noise related to the leaf surface. Thus, it created a bumpy surface and sometimes failed to generate parts of the surface, despite the presence of points in the cloud for those parts.

In contrast, our new method showed fewer artifacts (jagged outlines, twists, bumps, and missing surfaces), and thus, it represented an improvement compared with the two previously developed methods. The quantification of the degree of distortion showed that the proposed method extracts the distortion of the leaf shape under the range of conditions (Table 2) and throughout the growth sequence (Table 3). Note that although the model-free methods can complement the point cloud since it defines a mathematical model to explain the data beforehand, our method also has the ability to complement the point cloud since we define a simpler model of the leaf surface by the skeleton function. The rectangular area computed in the leaf area calculation can be seen as the simplest method to complement the occluded region in the point cloud using the skeleton.

In addition, we have added the leaf area computation result of the leaf surface reconstruction step of Zhu et al. [26] from their full process pipeline as the reference. The method from Zhu et al. [26] tended to contain many overestimates for both the soybean and sugar beet leaves (Figure 6, column 7). Zhu et al.'s method uses the LOESS method to fit the surface of the point cloud. The LOESS method potentially works well even with the presence of the discontinuity of points produced by heavy occlusions. Thus, it showed the best fit to our data in the model-free method we compared. However, it showed a high variation of leaf surface reconstruction when tested on time series data (Figure 7). It is probably caused by the edge computation part of the algorithm when applied to the leaf point cloud with noise and occlusion.

### 4.2. Potential for 3D Plant Phenotyping

The new leaf reconstruction method appears to be robust against noise and missing points in the point cloud despite our use of real-world data, and it worked well for species with different leaf shapes (Figure 6). The leaf area quantification was also more stable than the other model-free methods throughout the growth sequence (Figure 7 and Table 1), despite the noise, missing points, and dynamic changes in the leaf shape. This indicated that the proposed method could be used in practical 3D high-throughput plant phenotyping applications. Our method extracts the distortion that the leaf contains, so we could use the information to further extract the phenotypic characteristics. For example, skeleton points and the coefficient of the fitted function at the skeleton extraction process can be used as the leaf inclination degree or the leaf angle. In addition, because we were able to separately capture two components of the leaves (leaf shape and distortion), our method can track these characteristics of the leaf dynamically as the plant grows and develops. Thus, our method shows potential for supporting research on 3D leaf shape changes and in a field environment with a high-throughput and nondestructive way, under nonideal sensing conditions.

### 4.3. Limitations

Despite its strengths, our new method has certain limitations. First, it may not be able to account for sparse point clouds, because the proposed method splits the overall point cloud into strips, thereby reducing the number of data points available for each strip before it performs curve fitting for each strip. Although the cubic function chosen for the skeleton function can be used if there are at least three points per strip, a curve determined from only three points is unlikely to accurately represent the distortion of the leaf. Thus, the method's usefulness will improve as we increase the number of points available to determine the curve for each strip. We defined a threshold for the number of points in each strip's point cloud to mitigate this issue, but this results in some loss of surface points during the leaf flattening step in the rolling direction (Figure 6, rows A–C, column 7). Even when the strip contained more points than the threshold, some shape inaccuracy may develop due to the failure of the skeleton alignment during leaf flattening in the rolling direction. When the skeleton extraction from a strip's point cloud with very few points fails, alignment using the skeleton points fails. In this work, we performed the skeleton extraction of strips of the point cloud independent from the consecutive strip. We could use the information of the consecutive strip when the points are not enough. Second, our method cannot capture distortion that is more complex than the skeleton function. For example, we used a quadratic function to extract the shape distortion along the leaf's primary vein, but the quadratic function cannot model a shape that curls in two directions along that axis. However, because we can utilize any method that can successfully skeletonize the point cloud, it is possible to choose a different function for leaves with distinctive characteristics such as curls by applying other point cloud skeletonization techniques for plants or trees [36, 37]. Third, our method does not attempt to interpolate missing points. Although the proposed method is robust against missing points, it cannot infer the characteristics of regions that are obscured (e.g., by overlapping leaves). It is difficult to infer the occluded region from a single frame, but it might be possible when a sequence of point cloud data is utilized [38]. In addition, our method may not be applied to leaves with a particular type of shape, such as a lettuce leaf that reassembles a dragon curve surface or other heavily wavy leaves [39], since our method assumes leaves that become flat when placed on a flat table with one primary vein. We expect that these wavy leaves would consequently result in a strong underestimation of the actual surface area. A final challenge will be to analyze additional species with a wide range of leaf shapes and to analyze longer periods to monitor changes in phenology throughout the growing season to identify any limitations of the applicability of our method.

Since our method focuses on the leaf surface reconstruction step (Figure S1d), several problems are still needed to be solved in order to apply our method to the 3D plant phenotyping in actual use in the end-to-end pipeline. One of the essential issues is the segmentation of the individual leaf (Figure S1c). When the species that has the dense canopy structure (e.g., soybeans) is the research target, this issue becomes more challenging since the leaves overlap severely than the species that has the sparse structure (e.g., sugar beet). In the current study, the leaves have been segmented manually. We expect to make this process automated in the near future for practical high-throughput plant phenotyping applications.

## 5. Conclusion

In this study, we developed a new leaf surface reconstruction method that does not require a prior model of the leaf and is robust against noise and missing points. It therefore provides more stable and accurate leaf surface reconstruction than previous model-free methods (NURBS, Poisson, and LOESS methods). By capturing both the leaf shape and its distortion using specific properties of the leaves, the proposed method simplifies surface reconstruction compared with the previous methods, thereby improving its robustness. Because the proposed method is robust and stable despite point cloud data affected by noise and missing points, it shows great potential for practical high-throughput plant phenotyping applications. In future work, we will attempt to reconstruct a complete leaf surface without missing points, despite occlusion of some parts of the leaf surface in the input point cloud, and extend the method to other species as well.

## Figures and Tables

**Table 1:** Mean and standard deviation of the difference in leaf areas between consecutive frames from time series data of sugar beet leaves.

Leaf	NURBS low (cm^2^)	Poisson low (cm^2^)	Zhu et al. (cm^2^)	Proposed (cm^2^)
a	89.789 ± 721.427	0.865 ± 1.467	0.619 ± 1.098	0.306 ± 0.507
b	1092.003 ± 9179.370	0.993 ± 2.087	0.795 ± 2.533	0.302 ± 0.548

**Table 2:** Mean and standard deviation of the distance from the 2D projected plane for sugar beet and soybean leaves.

Species	Missing points	Leaf	*P* (input) (mm)	*P*′ (mm)	*P*′′ (mm)
Sugar beet	Few	a	3.784 ± 5.651	0.665 ± 0.833	0.211 ± 0.296
		b	2.490 ± 4.034	1.046 ± 1.472	0.292 ± 0.389
	Many	c	2.592 ± 3.406	1.756 ± 2.180	0.331 ± 0.445
		d	5.287 ± 6.727	2.277 ± 3.176	0.440 ± 0.722

Soybean	Few	e	1.579 ± 2.013	1.329 ± 1.719	0.505 ± 0.642
		f	2.470 ± 3.016	1.830 ± 2.198	0.576 ± 0.759
	Many	g	1.354 ± 2.620	1.352 ± 2.620	0.470 ± 0.631
		h	2.441 ± 3.173	2.364 ± 3.046	1.340 ± 2.767

**Table 3:** Mean and standard deviation of the distance from the 2D projected plane from time series data of sugar beet leaves.

Leaf	*P* (input) (mm)	*P*′ (mm)	*P*′′ (mm)
a	4.949 ± 7.063	1.484 ± 2.120	0.387 ± 0.664
b	4.450 ± 5.947	2.261 ± 3.186	0.504 ± 0.102

## Supplementary Materials

Supplementary Materials Figure S1: pipeline of the point cloud analysis of leaves. (a–e) Represent each step in the pipeline. Figure S2: overlapped leaf that is shown in Figure 6(d), G. Figure S3: zoomed examples of artifacts generated from the model-based methods from the results in Figure 6. Algorithm S1: pseudocode for the leaf axis determination step. Algorithm S2: pseudocode for the skeleton extraction process. Algorithm S3: pseudocode for the skeleton flattening process.
