# Supplementary material for: Robust Surface Reconstruction of Plant Leaves from 3D Point Clouds
Source: Plant Phenomics. 2021 Apr 2;2021:3184185. doi: 10.34133/2021/3184185 (PMC8038853; doi:10.34133/2021/3184185)
Supplement: Supplementary Materials — Figure S1: pipeline of the point cloud analysis of leaves. (a–e) Represent each step in the pipeline. Figure S2: overlapped leaf that is shown in Figure 6(d), G. Figure S3: zoomed examples of artifacts generated from the model-based methods from the results in Figure 6. Algorithm S1: pseudocode for the leaf axis determination step. Algorithm S2: pseudocode for the skeleton extraction process. Algorithm S3: pseudocode for the skeleton flattening process. [file 3184185.f1.docx]

Supplementary Materials


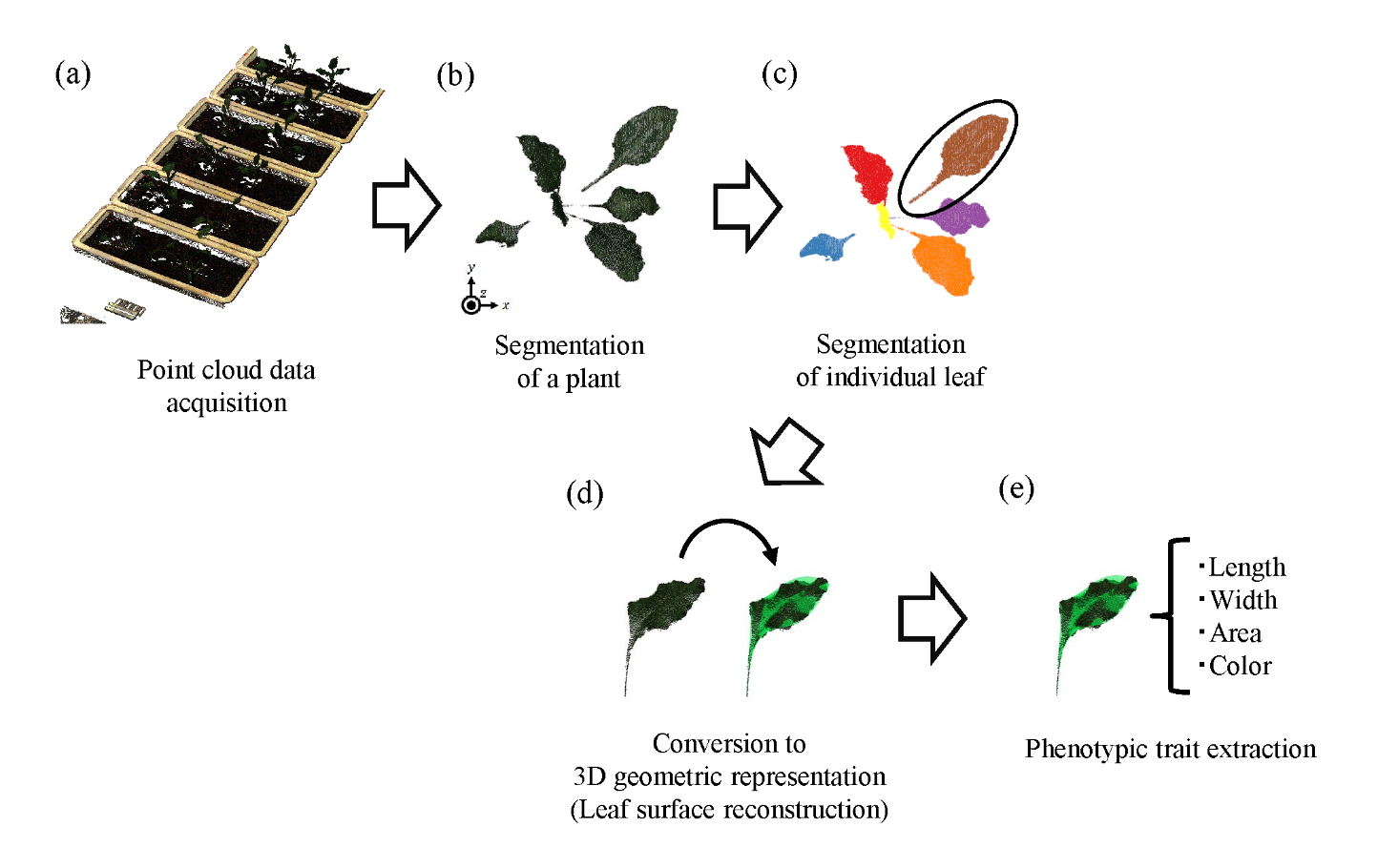


*Figure S1.* ***Pipeline of the point cloud analysis of leaf.*** *(a) to (e) represents each step in the pipeline.*


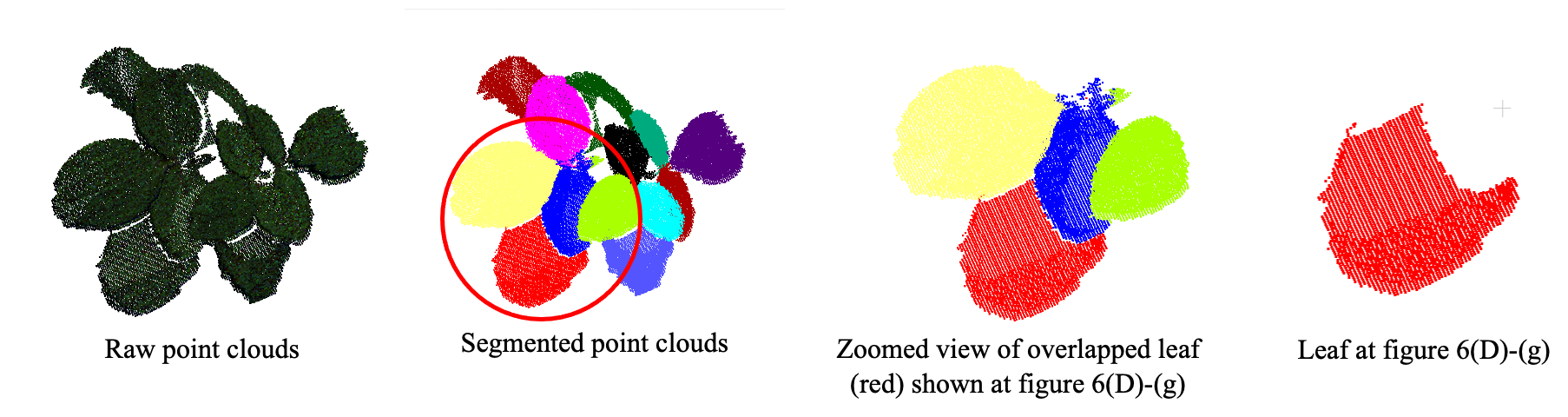


*Figure S2. Overlapped leaf that shown at figure 6(D)-(g).*


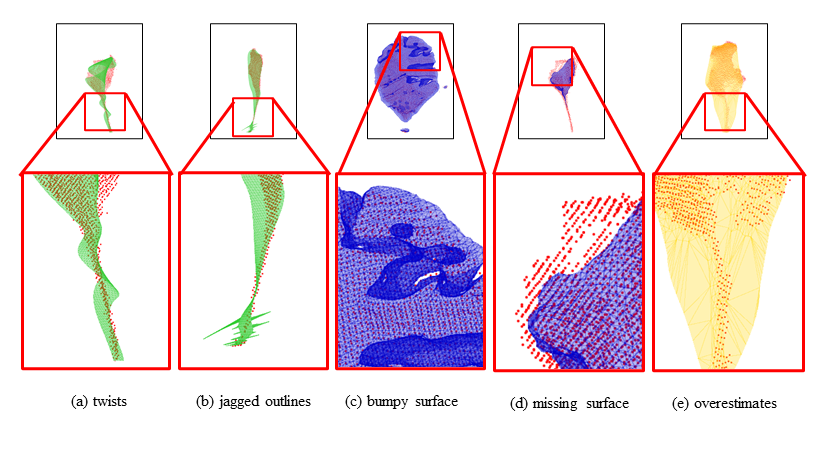


*Figure S3. Zoomed examples of artifacts generated from the model-based methods from the results in figure 6.*

*Algorithm S1.* ***Pseudo code for leaf axis determination step.***

| **Algorithm S1:** Leaf Axis Determination |
| --- |
| **Input:** the 3D leaf point cloud $P_{\mathrm{init}}$ |
| **Output:** Transformed 3D leaf point cloud $P$ |
| 1: Apply principal-components analysis to $P_{\mathrm{init}}$, and calculate $\boldsymbol{p}\boldsymbol{c}_{1}$, the eigenvector that corresponds to the largest eigenvalue of the covariance matrix for $P_{\mathrm{init}}$. |
| 2: Calculate the angle between $\mathbf{c}$**,** which is the centroid of $P_{\mathrm{init}}$, and $\boldsymbol{p}\boldsymbol{c}_{1}$. Determine the unit direction vector of the $l$-axis. |
| 3: Shift the origin of $P_{init}$ to $\boldsymbol{c}$ by calculating $P_{\mathrm{init}}\leftarrow P_{\mathrm{init}}-\boldsymbol{c}$. |
| 4: Generate an initial axis$d$ by selecting a random axis perpendicular to $l$-axis. |
| 5: Let $h_{\theta}$ be the axis that rotated the $d$-axis ($h_{\theta=0}=$ $d$) around the $l$-axis for the angle $\theta$.  Let $I_{h_{\theta}l}$ be the binary image generated by creating a grid over $P_{\mathrm{init}}$ projected onto the $h_{\theta}l$-plane. The pixel value is 1 if a point exists inside the grid cell or otherwise 0.  Let $S\left( \theta\right)$ be the sum of the pixel values of binary image $I_{h_{\theta}l}$. |
| 6: Compute $\theta^{'}=\underset{\theta}{arg min} S(\theta)$ for $\theta=0^{\circ}, 1^{\circ}, \cdots, 180^{\circ}$ and set a unit direction vector of the $h_{\theta^{'}}$-axis as the $h$-axis direction vector $\boldsymbol{h}$. |
| 7: Determine whether the $z$-axis element of $\boldsymbol{h}$ points towards the ground or not. Flip the $z$-axis 180° if it does. |
| 8: Determine the $w$-axis by computing the cross product of $\boldsymbol{l}$ and $\boldsymbol{h}$. |
| 9. Transform $P_{\mathrm{init}}$ from the original ($x, y, z)$ coordinate system to the ($l, w, h)$ leaf coordinate system to compute $P$. |

*Algorithm S2.* ***Pseudo code for skeleton extraction process.***

| **Algorithm S2:** Skeleton Extraction |
| --- |
| **Input:** 3D leaf point cloud $Q$ ($\delta\leq M$) |
| **Output:** Skeleton points $S$ |
| 1: Project $Q$ onto the $ab$-plane that contains the skeleton. |
| 2: Fit a skeleton function $f$ by means of least-squares linear regression. |
| 3: Create the leaf point cloud $U$ by sampling $N$ points uniformly between the minimum and maximum values of the $a$-axis elements of the points from the projected $Q$. |
| 4: Compute skeleton points $S$ by $S=f\left( U \right)$. |

*Algorithm S3.* ***Pseudo code for skeleton flattening process.***

| **Algorithm S3:** Skeleton Flattening |
| --- |
| **Inputs:** 3D leaf point cloud $Q$, skeleton points $S=\left\{ \boldsymbol{s}_{i}\in\mathbb{R}^{2} \vert0\leq i\leq N-1 \right\}$ |
| **Output:** Flattened leaf point cloud $Q'$, Flattened skeleton points $S^{'}=\left\{ \boldsymbol{s'}_{i}\in\mathbb{R}^{2} \vert0\leq i\leq N-1 \right\}$ |
| 1: Initialize by setting $\boldsymbol{s'}_{0}=\left( 0, 0 \right)$. |
| 2: **for** $k=0, 1, \cdots, N-2$ **do**: |
| 3: Calculate the angle $\varphi$ between the $k+1$th skeleton point $\boldsymbol{s}_{k+1}$ and the $a$-axis  (Fig. 4a). |
| 4: Compute ${\boldsymbol{s}^{\boldsymbol{'}}}_{k+1}$ by rotating $\boldsymbol{s}_{k+1}\boldsymbol{-}\boldsymbol{s}_{k}$ for $\varphi$ around the origin of the $ab$-plane and translated it along the $a$-axis by distance $t_{k}$ (Fig. 4b). |
| 5: Project $Q$ onto the $ab$-plane to generate $Q^{ab}$, rotate it for $\varphi$ around the origin of the $ab$-plane, and then translate it along the $a$-axis by distance $t_{k}$ to compute 2D point cloud $V$ (Fig. 4c). |
| 6: From $V$, extract points that exist in between the skeleton points $\boldsymbol{s}_{k}$ and $\boldsymbol{s}_{k+1}$ to generate the 2D point cloud $V^{\left( k \right)}$. Concatenate the last element of $Q$ that is not used for the skeleton plane to $V^{\left( k \right)}$ and generate a 3D strip leaf point cloud $Q^{(k)}$ (Fig. 4d). |
| 7: **end for** |
| 8: Gather $Q^{(k)}$ for every $k$ to obtain the flattened skeleton leaf point cloud $Q'$ (Fig. 4e)). |
